# A systematic review on diagnostics and surgical treatment of adult right-sided Bochdalek hernias and presentation of the current management pathway. Author’s reply

**DOI:** 10.1007/s10029-022-02647-1

**Published:** 2022-06-18

**Authors:** J. P. Ramspott, M. Lechner, T. Jäger, K. Emmanuel, S. Regenbogen, the Working Group of Surgical Oncology (WOGSO)

**Affiliations:** 1 Department of Surgery, Paracelsus Medical University Salzburg, Salzburg, Austria; 2 Department for General, Visceral and Transplant Surgery, University Hospital Muenster, Waldeyerstraße 1, 48149 Muenster, Germany; 3 Department of Trauma Surgery, BG Trauma Center Murnau, Murnau, Germany

Dear Editor,

We would like to thank Tzortzis [1] for his interest in our recent systematic review [2] and we appreciate the opportunity given by the HERNIA Journal to respond to his comments.

To clarify the difference in the utilization rates of minimally invasive techniques in elective versus emergency settings as well as suture versus mesh repair we once again analyzed our data.

Our systematic review identified 41 studies describing 44 cases of right-sided Bochdalek hernias in adulthood [2]. Only 19 cases (43%) gave information about the type of surgery (emergency surgery or not, no surgery), but they did not define what “emergency” means in the individual case.

Interestingly, all emergency surgeries (*n* = 7, 16%) were performed via laparotomy and in five of seven cases direct diaphragmatic suture was performed. Two emergency cases did not report the type of defect closure. Three cases (7%) underwent small bowel resection and in one case colon resection was performed whereas three cases did not describe if bowel resection was necessary.

In only four of twelve non-emergency cases (9%), laparoscopy was performed. In two cases (5%), right-sided double-J ureteral stent due to preoperative hydronephrosis and percutaneous nephrostomy due to patient’s fitness was performed. Further details were not described. Three non-emergency cases (7%) were treated with open surgery (direct diaphragmatic suture in two cases and mesh in one case). Three patients (7%) underwent no intervention. In most non-emergency cases (*n* = 8, 18%) bowel resection was not necessary.

As already mentioned in our systematic review, most studies investigating the management of adult non-traumatic right-sided Bochdalek hernias are of moderate-to-low methodological quality. Most studies did not give any information whether emergency surgery was necessary or not. Therefore, based on the most recent available data, we are uncertain whether or not ‘rules of thumb’ can be developed and we had difficulties in correlating some parts of the data cited by Tzortzis [1] to the mentioned literature.

The question of open versus laparoscopic surgery cannot be answered on a general basis. Both approaches appear feasible based on the available literature, but in general, the decision is also based on the patient’s overall condition and the surgeon’s laparoscopic and thoracoscopic experience. Similarly, the use of direct suture, mesh implant, or the combination of both depends on the type of presentation, parameters, such as defect size, and the potential presence of concomitant (fecal) contamination. Finally, the term ‘complicated hernia’ can encompass any or all of the mentioned circumstances and different levels of surgical expertise may make a relevant difference in what is seen as a complicated hernia and what is not.

Based on these considerations and the available literature, it was our aim to formulate a management pathway that takes these circumstances into account and which highlights the principles of quick diagnosis, urgent repair of complicated hernias (i.e., acutely incarcerated or strangulated hernia with or without hollow viscus organ perforation), the simultaneous exploration and de-contamination of the pleural cavity, and last but not least the need for scheduled long term follow-up.

We appreciate again for the comments on our study and hope that our explanations will help our readers to clarify our data.
